# Reconstruction of a Subtotal Maxillectomy Defect Using a Customized Titanium Implant in a 4-Year-Old Child: An 8-Year Follow-Up Report

**DOI:** 10.3389/fsurg.2020.00028

**Published:** 2020-06-02

**Authors:** Maurice Yves Mommaerts

**Affiliations:** European Face Centre, Universitair Ziekenhuis Brussel, Vrije Universiteit Brussel, Brussels, Belgium

**Keywords:** titanium, follow-up studies, craniotomy, facial bones, paranasal sinuses

## Abstract

**Condition:** This case report demonstrates the use of alloplastic reconstruction in young children.

**Method:** A three-dimensionally printed titanium implant was used to reconstruct a subtotal maxillectomy defect in a 4-year-old child.

**Results:** We report an 8-year follow-up. The endoprosthesis was split at the midline to address transverse growth. The main finding is that the stigma surrounding resection and surgical reconstruction in pre-adolescents can be prevented by the use of alloplastic reconstruction based on titanium osseointegration. An additional finding is that shear forces should be prevented at the insertion points of the fixation screws in the facial walls by providing a vertical support for the maxillary/palatal shelves. Lastly, transverse maxillary growth in the circumferential sutures and functional matrix was not hampered by splitting the endo- and exoprostheses in the middle (where the mid-palatal suture would normally be located).

**Conclusion:** Alloplastic reconstruction of maxillectomy defects in childhood can offer a viable temporary solution.

## Introduction

In 1999, Peckitt reported a case involving the flapless repair of a low-level total maxillectomy defect using a computer-numeric, control-milled titanium alloy implant. Zygomatic flanges were welded onto the implant body to attach it to the zygomata (1). An overdenture was preoperatively constructed and held in place by Nobel Biocare SDCB 116 abutments. A stereolithographic model was used to determine the resection margins, and a class II workflow was used to design and manufacture the implant (2). In this case, the 76-year-old patient was also diagnosed with squamous cell carcinoma.

Here, we report on the use of a similar approach in a 4-year-old girl, presenting with aggressive juvenile fibromatosis of the anterior maxilla.

## Case Report

**Figure 1:**
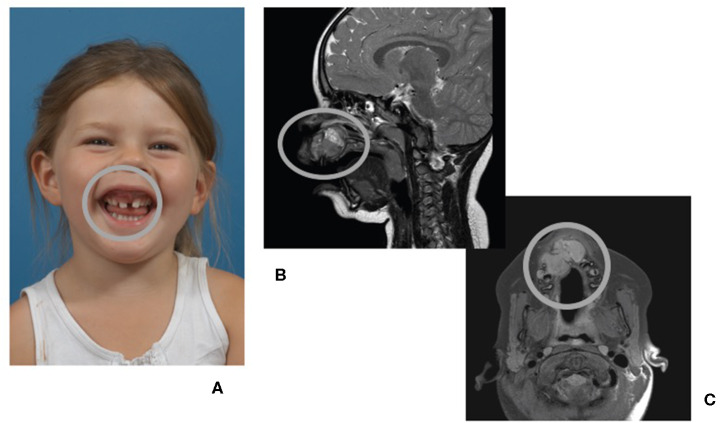
A 4-year-old child presenting with a fast growing tumor (circled in all panels) of the anterior maxilla. **(A)** Front view of patient, smiling. **(B)** Magnetic resonance imaging (MRI) of the head, midsagittal plane. **(C)** MRI of the head, coronal plane.

**Figure 2:**
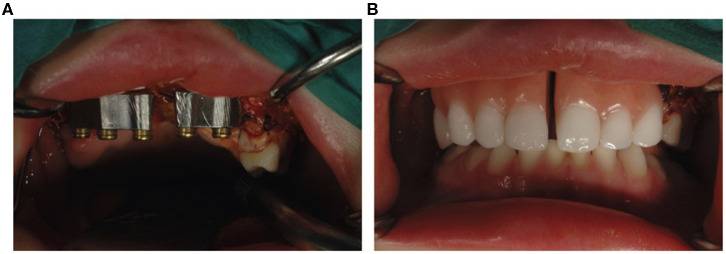
Double-structure denture fixed on two subperiosteal implants. **(A)** Primary structure with LOCATOR precision attachments. **(B)** Split overdenture.

A child who was born on April 11, 2007 presented with a mass that developed over a 2-month period (Figure 1) prior to the initial consultation. She received curative resection of aggressive juvenile fibromatosis on July 13, 2011, after the proper diagnostic work-up. After the resection of two-thirds of her maxilla, a conventional obturator that was suspended from the zygomatic arches by wires was used for initial wound healing. One month later, the cone-beam computer tomography dataset of the cranium was reverse-engineered to design the zygomatic shelves (Materialise NV, Heverlee, Belgium), connected to a double-structure denture (Elysée Dental NV, Leuven, Belgium) that had been biomechanically tested (Mobelife NV, Leuven, Belgium). The left and right implants were additively manufactured in titanium (Layerwise Inc., Leuven, Belgium) and paired with LOCATOR precision attachments (Figure 2). The soft tissue of the cheek underwent submucosal dissection, and the midline was approximated. The posts of the endoprosthesis were not covered (single stage surgery). The endo- and exoprostheses were positioned on September 5, 2011. Partial dehiscence occurred soon after the first prosthesis was placed, and the size of the resulting oronasal fistula increased over time. During future procedures, the fistula was obstructed by the palatal flange of the replacement exoprosthesis.

**Figure 3:**
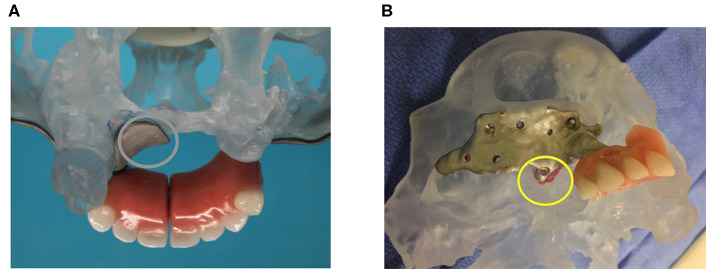
The two subperiosteal implants. **(A)** Posterior view of the stereolithographic defect model with the left subperiosteal implant abutting vertically on the palatal shelf. **(B)** Right subperiosteal replacement implant, now also abutting vertically (yellow circle), albeit on a very small portion of the maxilla.

One year later, the right implant loosened and was replaced by a new one on February 4, 2013 (Figure 3). This loosening was likely due to the shear forces and stress on the fixation screws, which were not prevented by a vertical support. The replacement implant was designed to incorporate such features (Figure 3B).

However, proper hygiene proved difficult over the first 2 years. Inflammation was anticipated because proper oral hygiene in a child who had recently undergone surgery was difficult to maintain. Occasional inflammatory edema of the cheek and pus around the right post were noted. With professional cleaning and increased self-motivation, the child was able to avoid these uncomfortable periods beginning 2015.

**Figure 4:**
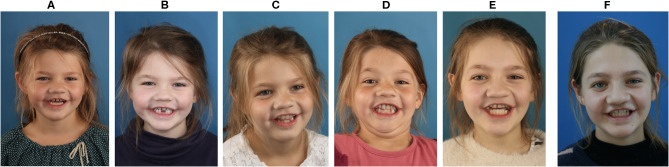
Front views of patient, smiling, during the years **(A)** 2014, **(B)** 2015, **(C)** 2016, **(D)** 2017, **(E)** 2018, and **(F)** 2019. Flaws in the vertical midface and differences in the distance between the top and bottom eyelids have become apparent during the secondary growth spurt.

The patient had the secondary structure containing artificial front teeth replaced four times over 6 years (2015, 2016, 2018, and 2020) because of the development of an anterior cross-bite (Figure 4). Moderately deficient sagittal and vertical growth was observed. However, transverse occlusion in the posterior dentate jaw segment was maintained. The patient adhered to and tolerated the interventions well. The development of a frontal cross-bite due to a sagittal growth impairment was anticipated. The patient appears confident and has no issues except for the alignment and color of the fabricated artificial denture.

## Discussion

Literature regarding maxillectomy defect reconstruction using prostheses fixed on subperiosteal frames is sparse. Of the three case presentations of uni-/bilateral maxillectomy for oral squamous cell carcinoma and bilateral maxillectomy/partial midface resection for a malignant peripheral nerve sheath tumor, radiotherapy was mentioned in only two (3–5). One patient had received radiotherapy 7 years prior to reconstruction surgery and was followed up for 6 months. The soft tissue remained in good condition (4), despite radiotherapy being known to decrease the success rate for root-shaped endosseous (6) and zygomaticus implants (7). Furthermore, implants may even trigger osteonecrosis. For this reason, we do not recommend the routine use of additively manufactured subperiosteal jaw implants (AMSJIs) (8, 9) for oncology cases in which radiotherapy is involved. Unfortunately, these patients would otherwise benefit greatly from this patient-specific solution.

Treatment of maxillofacial tumors at a young age poses challenges of growth disturbances, no matter which treatment modality is applied (10). In this patient, transverse growth occurred as expected, likely because a split implant and denture approach was chosen. This approach allowed for growth in the facial sutures, including the transverse growth in the remaining posterior mid-palatal suture. Both sagittal and vertical appositional growth were hampered by the resection, and the development of anterior dentoalveolar structures was absent. Interestingly, the distance increased between the upper and lower eyelids on the right side, and the cheek soft tissues on the right side appeared bulkier.

Reconstruction using a three-dimensionally printed subperiosteal implant after subtotal maxillary resection in the presented case was preceded by total mandibular replacement with a three-dimensionally printed endoprosthesis 1 month earlier (the latter case was never published in scientific literature).

## Conclusion

Temporary reconstruction using three-dimensionally printed titanium subperiosteal implants is an attractive option in young children. The closure of soft tissue defects using local flaps is often difficult in subtotal maxillary resections. Locoregional and pedicled flaps may damage the face or other visible regions of the neck and/or torso. The transfer of microvascular bony tissue can be postponed until the completion of growth, when vessel size is less critical. After another 6 years, it remains to be determined whether bony reconstruction will remain the gold standard for reconstruction or whether subperiosteal implants including facial contouring and fixation of a hybrid bridge will provide an alternative method.

## Data Availability Statement

All datasets generated for this study are included in the article/Supplementary Material.

## Ethics Statement

Ethical approval was not required. The parents consented in writing to the publication of this case report, including the facial pictures.

## Author Contributions

MM supervised the design and implanted the endoprosthesis, followed-up on the case, drafted, and corrected the manuscript.

## Conflict of Interest

The author declares that the research was conducted in the absence of any commercial or financial relationships that could be construed as a potential conflict of interest.
